# 4‐phenylpyridine suppresses UVB‐induced skin inflammation by targeting c‐Src in vitro and in vivo

**DOI:** 10.1111/jcmm.17422

**Published:** 2022-06-10

**Authors:** Ju Gyeong Kim, Ha Yeong Kang, Min Jeong Kim, Seokwon Lim, Chang Joo Lee, Kyung‐Min Kim, Sung Keun Jung

**Affiliations:** ^1^ School of Food Science and Biotechnology Kyungpook National University Daegu Korea; ^2^ Department of Food Science and Biotechnology Gachon University Seongnam‐si, Gyeonggi‐do Korea; ^3^ Department of Food Science and Biotechnology Wonkwang University Iksan Korea; ^4^ School of Applied Biosciences Kyungpook National University Daegu Korea; ^5^ Research Institute of Tailored Food Technology Kyungpook National University Daegu Korea

**Keywords:** 4‐phenylpyridine, *Brassica campestris* L. ssp. *pekinensis*, COX‐2, c‐Src, phytochemicals, skin inflammation

## Abstract

Acute or repetitive exposure to ultraviolet (UV) cause disruptions to the skin barrier and subsequent inflammatory skin disease. 4‐phenylpyridine (4‐PP) is a constituent of *Brassica campestris* L. ssp. *Pekinensis* and its effect on skin inflammation and molecular target remain unclear. The purpose of this study is to confirm the anti‐inflammatory efficacy of 4‐PP on UVB‐induced skin inflammation in human keratinocytes HaCaT and mouse skin and validation of its molecular target. 4‐PP also attenuated UVB‐induced phosphorylation of p38/mitogen‐activated protein kinase kinase (MKK) 3/6, c‐Jun N‐terminal kinase 1/2, MKK 4/7, extracellular‐signal‐regulated kinase 1/2, mitogen‐activated protein kinase 1/2. Additionally, 4‐PP inhibited UVB‐induced phosphorylation of epidermal growth factor receptor (EGFR) Y1068, Y1045 and 854 residues but not the proto‐oncogene tyrosine‐protein kinase c‐Src. Drug affinity responsive target stability assay revealed that 4‐PP directly binds to c‐Src and inhibits pronase c‐proteolysis. Knockdown of c‐Src inhibited UVB‐induced COX‐2 expression and phosphorylation of MAPKs and EGFR in HaCaT cells. Dorsal treatment of 4‐PP prevented UVB (0.5 J/cm^2^)‐induced skin thickness, phosphorylation of EGFR and COX‐2 expression in mouse skin. Our findings suggest that 4‐PP can be used as anti‐inflammatory agent with an effect of skin inflammation by inhibiting the COX‐2 expression via suppressing the c‐Src/EGFR/MAPKs signalling pathway.

## 1 INTRODUCTION

Skin acts as the primary defence organ and is composed of epidermis, dermis and subcutaneous tissue^1^ that protects against ultraviolet (UV) irradiation, environmental toxins, allergens, pathogens, dehydration and other external threats.^2^ Disruption of the skin barrier is associated with the development of inflammatory skin disease including atopic dermatitis, psoriasis, rosacea and skin cancer.^3^ Although UV irradiation can have beneficial effects, such as vitamin D synthesis and killing pathogens, acute and chronic UV exposure to human skin causes skin inflammation, photoageing and skin cancer.^4^ UV light is divided into three components of different wavelengths: UVA (320–400 nm), UVB (280–320 nm) and UVC (200–280 nm). UVB is considered as carcinogenic as exposure to these wavelengths alone resulted in skin cancer in SKH‐1 hairless mice.^5^

COX‐2 is an inducible enzyme that catalyses the rate‐limiting conversion of prostaglandin E2 (PGE_2_), a critical mediator in inflammation.^6^ UVB‐induced COX‐2 has a vital role in skin inflammation and cancer. Celecoxib, a COX‐2 inhibitor, suppressed UVB‐induced skin inflammation such as skin thickness, PGE_2_ production, sunburn and carcinogenesis in vivo.^7^ Inhibition of COX‐2 expression increased by UV irradiation also suppressed the development of skin inflammation, photoageing and skin cancer.^8^ Signalling molecules mitogen‐activated protein kinases (MAPKs) and activated protein (AP)‐1 transcriptional factor directly regulate UVB‐induced COX‐2 expression in HaCaT and SKH‐1 hairless mice.^5^ Although the cell receptor for UV has not been fully elucidated, epidermal growth factor receptor (EGFR) links UV‐induced MAPKs phosphorylation and AP‐1 activity. A previous study reported that after UVB radiation, c‐Src encourages the internalization of active EGFR, which in turn leads to the activation of the ERK1/2 pathway, resulting in increased COX‐2 expression.^9^ The c‐Src inhibitor, PP2, inhibited UVB‐induced COX‐2 expression and phosphorylation of ERK, p38, c‐Jun N‐terminal kinase (JNK), Akt and EGFR (Y845) compared with scrambled control in JB6P+ cells.^10^ Therefore, inhibition of c‐Src and EGFR signalling pathway could be a therapeutic target to alleviating UV‐induced skin inflammation.

Botanical extracts are frequently used as traditional remedies for inflammation. Anti‐inflammatory effects of extracts and phytochemicals in fruits, herbs and vegetables have been reported in skin‐related cells and mice.^11^ 4‐phenylpyridine (4‐PP) is a volatile compound present in *Brassica campestris* L. ssp. *pekinensis* known as Chinese cabbage and/or Korean cabbage.^12^ Although it is reported that Chinese cabbage exerts antioxidant, antimicrobial and antiatherosclerosis properties.^13^, ^14^ the effect of 4‐PP on UVB‐induced skin inflammation and its molecular target(s) remain unclear.

Here, we investigated the effect of 4‐PP on UVB‐induced COX‐2 expression and skin inflammation in HaCaT human keratinocytes and ICR mice. We confirmed that 4‐PP suppressed UVB‐induced *COX‐2* gene expression in HaCaT cells and COX‐2 expression in vitro and in vivo. Furthermore, 4‐PP suppressed UVB‐induced c‐Jun phosphorylation and translocation from cytosol to the nucleus by suppressing EGFR/MAPKKs/MAPKs signalling pathways via direct binding to c‐Src. Based on these results, we suggest that 4‐PP may be a potent anti‐inflammatory agent that inhibits COX‐2 expression through direct binding to c‐Src.

## 2 MATERIALS AND METHODS

### 2.1 Materials

4‐phenylpyridine was purchased from Sigma Aldrich (Sigma Aldrich, St. Louis, USA). Dulbecco's Modified Eagle Medium (DMEM), penicillin–streptomycin and fetal bovine serum (FBS) were from Thermo Scientific HyClone (Thermo Scientific HyClone, Logan, USA). Antibodies to β‐actin (1:1000, sc‐517,582) and c‐Src (B‐12) (1:1000, sc‐8056) were purchased from Santa Cruz Biotech (Santa Cruz Biotechnology, CA, USA). Antibodies specific for COX‐2 (1:1000, #12282), c‐Jun (1:1000, #9165), p‐c‐Jun (1:1000, #3270), α/β‐tubulin (1:1000, #2148), p38 MAPK (1:1000, #8690), phospho‐p38 MAPK (Thr180/182) (1:1000, #4511), SAPK/JNK (1:1000, #9252), phospho‐SAPK/JNK (Thr183/Tyr185) (1:1000, #4668), p44/42 MAPK (Erk1/2) (1:1000, #4695), phospho‐p44/42 MAPK (Erk1/2) (Thr202/Thr204) (1:1000, #4370), p‐Akt (S473) (1:1000, #9271), Akt (1:1000, #9272), MKK3/6 (1:1000, #9238), p‐MKK3/6 (1:1000, #9231), MKK4/7 (1:1000, #9152), p‐MKK4/7 (1:1000, #9156), MEK1/2 (1:1000, #9122), p‐MEK1/2 (1:1000, #9121), p‐Src (Y416) (1:1000, #6943), p‐EGFR (Y845) (1:1000, #2231), p‐EGFR (Y1068) (1:1000, #3777), p‐EGFR (Y1045) (1:1000, #2237) and EGFR (1:1000, #4267) were purchased from Cell Signalling Biotechnology (Cell Signalling Biotechnology,). Anti‐lamin B1 (1:10000, ab 16,048) was purchased from Abcam (Abcam).

### 2.2 Cell culture, UVB exposure and cell viability

Human epidermal keratinocyte HaCaT cells were cultured in DMEM containing 10% FBS, and 1% penicillin–streptomycin at 37°C and 5% CO_2_ in a humidified incubator (Thermo Fisher Scientific,).

UV Bio‐link crosslinker (Vilber Lourmat,) and UVB lamp (Vilber Lourmat,) with wavelength of 312 nm were used for UVB irradiation; HaCaT cells were irradiated with UVB at 0.03 J/cm^2^.

HaCaT cells were grown to 70%–80% confluency in a 96‐well plate and pretreated with 4‐PP dissolved in dimethyl sulfoxide (Sigma Aldrich,) at 12.5, 25, 50 μM for 24 h; 3‐(4,5‐dimethylthiazol‐2‐yl)‐5‐(3‐carboxymethoxyphenyl)‐2‐(4‐sulphophenyl)‐2H‐tetrazolium (MTS) was added with phenazine methosulphate (Promega,). Cell viability was measured with a microplate reader at an absorbance of 490 nm (Bio‐Rad Inc.,).

For in vitro study, we replaced with serum free before sample treatment, cultured for 24 h, and then conducted the further experiments.

### 2.3 Animal experiments

5‐weeks‐old male ICR mice were purchased from Joongah Bio (Joongah Bio,). The animals were housed in climate‐controlled quarters (25°C at 50% humidity) with a 12‐h light/12‐h dark cycle. All animals received humane care, and the study protocol (2020–0007) was approved and performed in accordance with guidelines for animal use and care at Kyungpook National University. Animals were stabilized for 1 week prior to the study with free access to food and water. For animal study, acetone was used as vehicle and 4‐PP was dissolved in acetone. Twenty mice were randomly allocated to each group (*n* = 5 per each group, four groups in total): (1) control group (normal), (2) UVB‐irradiated group (UVB), (3) 0.2 mM 4‐PP treated and UVB‐irradiated group, and (4) 1 mM 4‐PP treated and UVB‐irradiated group. The day before the experiment, mice were treated with 200 μl of 4‐PP after hair removal. On the day of the experiment, on the back of the mice were topically treated with 4‐PP for 1 h before UVB exposure (0.5 J/cm^2^). After 4 h, mice were sacrificed, and then dorsal skin was separated.

### 2.4 PGE_2_ enzyme‐linked immunosorbent assay

HaCaT cells (1 × 10^5^ cells/ml) were grown to 70%–80% confluency in a 6 cm dishes. The media was replaced with serum‐free DMEM for 24 h and 4‐PP added at 12.5, 25 and 50 μM. After incubating for 1 h, HaCaT cells were irradiated with UVB (0.03 J/cm^2^) and incubated for 20 h. PGE_2_ released into the medium was measured using a PGE_2_ enzyme immunoassay kit (R&D Systems Inc.,) according to manufacturer's instructions. Absorbance values at 450 nm were measured using a microplate reader (iMark™ Microplate Reader; Bio‐Rad Laboratories).

### 2.5 Reverse transcriptase quantitative PCR

HaCaT cells (1 × 10^5^ cells/ml) were grown to 70%–80% confluency in 10 cm dishes. The media was replaced with serum‐free DMEM for 24 h and 4‐PP added at 12.5, 25 and 50 μM. After incubating for 1 h, HaCaT cells were irradiated with UVB (0.03 J/cm^2^) and incubated for 4 h. Total RNA was extracted using an RNA isolation buffer (TaKaRa,) and converted into cDNA by reverse‐transcription with the ReverTra® Ace qPCR Rt Master Mix (TOYOBO,) using thermal cycler (TaKaRa,) at 37°C for 15 min, 95°C for 5 min. The target gene was amplified using 20 μl SYBR green real‐time PCR master mix (TOYOBO), 0.05 μg of cDNA and following primers: human *COX‐2* Fw‐5′‐GAATCATTCACCAGGCAAATT‐3′ and Rv‐5′‐TTTCTGTACTGCGGGTGGAAC‐3′, and human *β‐actin* Fw‐5′‐CCTCACCCTGAAGTACCCCA‐3′ and Rv‐5′‐TGCCAGATTTTCTCC ATGTCG‐3′). Relative gene expression was determined on real‐time PCR detection system (Bio‐Rad Inc., Hercules, CA, USA) using the comparative ΔΔCq method and the housekeeping gene *β‐actin* to normalize the data.

### 2.6 AP‐1 promoter assay

HaCaT cells (1 × 10^5^ cells/ml) stably infected with pGreeFire plasmid containing MMP‐1 promoter were grown to 70%–80% confluency in 96‐well plates. The media was replaced with serum‐free DMEM for 24 h and 4‐PP (12.5, 25 and 50 μM) added for 1 h. After UVB (0.03 J/cm^2^) irradiation, cells were incubated for 5 h. Cells were disrupted with lysis buffer [0.1 M potassium phosphate buffer [pH 7.8], 1% Triton X‐100, 1 mM dithiothreitol (DTT), and 2 mM ethylenediaminetetraacetic acid (EDTA)], and luciferase activity was measured on luminometer (Promega,).

### 2.7 Western blot assay

For in vitro Western blot assays, HaCaT cells (1 × 10^5^ cells/ml) were grown to 70%–80% confluency in 10 cm dishes. The media was replaced with serum‐free DMEM for 24 h and 12.5, 25 and 50 μM 4‐PP added for 1 h. After irradiation UVB (0.03 J/cm^2^), cells were incubated for a specific time period. The cells were washed twice with phosphate‐buffered saline (PBS) and then collected with cell lysis buffer (Cell Signalling Biotechnology,). Cell lysate was maintained on ice for 30 min and centrifuged at 4°C, 13,652 × *g* for 15 min.

For in vivo western blot assays, mice were sacrificed, and then mouse skin was separated. Mouse skin tissue was added to 2 ml microcentrifuge tubes containing lysis buffer and stainless‐steel beads and subsequently homogenized using a Precellys 24 dual homogenizer tissue homogenizer (Bertin, Montigny‐le‐Bretonneux). Mouse skin lysates were centrifuged at 4°C, 13,652 × *g* for 15 min.

Protein concentration of separated supernatant was measured using DC™ Protein Assay kit (Bio‐Rad Inc.,). Cell lysates or skin tissue extract were mixed with 5× sodium dodecyl sulphate‐polyacrylamide gel electrophoresis (SDS‐PAGE) loading buffer (Biosesang,) and separated by 10% SDS‐PAGE. Polyvinylidene fluoride membrane (Millipore, Immobilon®‐P transfer membrane) transferred protein was incubated with a specific primary antibody at 4°C overnight. After incubation with the secondary antibody, the protein bands were detected with a chemiluminescence detection kit (ATTO,) using Chemiluminescence Systems instrument (SYNGENE,). The band intensity of western blot was analysed using image J software program (National Institutes of Health,).

### 2.8 Cytosolic and nuclear fractions

HaCaT cells (1 × 10^5^ cells/ml) were grown to 70%–80% confluency in 10 cm dishes. The media was replaced with serum‐free DMEM for 24 h and 4‐PP added at 12.5, 25 and 50 μM for 1 h. After UVB irradiation (0.03 J/cm^2^), cells were incubated for 30 min, collected with PBS and separated with the NE‐PER™ Nuclear and Cytoplasmic Extraction Reagent (Thermo Fisher Scientific,). Cell extracts separated into nucleus and cytoplasm were assessed via western blot assay.

### 2.9 Immunofluorescence assay

For in vitro immunofluorescence assay, HaCaT cells (1 × 10^5^ cells/ml) were grown to 50%–70% confluency in 8‐well chambers (ibidi,). Media was replaced with media plus 4‐PP at 12.5, 25 and 50 μM for l h. After UVB irradiation (0.03 J/cm^2^), cells were incubated for 15 min.

For in vivo immunofluorescence assay, mouse skins separated from back were embedded with OCT solution (Leica Biosystems Richmond Inc.,) in frozen section. Frozen section was cut into 6‐μm‐thick sections under a microscope (Cryostat CM1850, Leica Biosystems,) and then attached to microscope slides (Thermo Fisher Scientific,).

Cells and frozen tissues were fixed with 4% formaldehyde and permeabilized with ice‐cold 100% methanol. After blocking, cells were incubated with specific antibodies at 4°C overnight. Goat anti‐rabbit IgG H&L conjugated to Alexa Fluora® 488 secondary antibodies (Abcam) were incubated with the cells for 1–2 h. Nuclei were counterstained with DAPI antibody (VECTASHIELD®: Vector Laboratories, Burlingame, CA). The location of c‐Jun was determined using a confocal laser scanning microscope (Carl Zeiss Co Ltd.,).

### 2.10 Drug affinity responsive target stability assay

DARTS assay was performed as previously described (Lomenick et al., 2009). HaCaT cells (1 × 10^5^ cells/ml) were grown to 70%–80% confluency in a 10 cm dish. The media was replaced with serum‐free DMEM for 24 h. Cells were washed twice with PBS and then collected with M‐per lysis buffer (Thermo Fisher Scientific, Waltham**,** USA). Cell lysate was maintained on ice for 10 min and centrifuged at 4°C, 18,000 × *g* for 15 min. 10× TNC buffer was added to the lysates, and the protein concentration quantified using DC™ Protein Assay kit by describing at Western blot assay. Dimethyl sulfoxide and 4‐PP were added to the lysate and incubated for 1 h at room temperature. The lysate was treated with pronase for 30 min, and then assessed via western blot assay.

### 2.11 Small interfering RNA knockdown assay

HaCaT cells were grown to 50%–60% confluence in 10 cm dishes. Cells were transfected with control‐scrambled siRNA and Src siRNA duplex (RNA‐CUC UAU GAC UAU GAG UCU A = tt(1‐AS) and RNA‐UAG ACU CAU AGU CAU AGA G = tt(1‐AA)) with lipofectamine 2000 (Invitrogen,). Transfected cells were used for the experiments after 24 h of additional incubation in opti‐MEM medium (Thermo Fisher Scientific,). After irradiation UVB (0.03 J/cm^2^), cells were incubated for a specific time period, and then assessed via western blot assay.

### 2.12 Histological analysis

Mouse skin separated from back was embedded with OCT solution in frozen section. Frozen section was cut into 6‐μm‐thick sections under a microscope (Cryostat CM1850, Leica Biosystems, ) and then attached to microscope slides (Thermo Fisher Scientific,). For histological analysis, sections were stained 3% haematoxylin solution (Sigma) for 5 min and then rinsed in cool running ddH_2_O for 5 min. Sections were dipped in 0.5% eosin (Sigma) solution and then rinsed in cool running ddH_2_O for 5 min. Subsequently, sections were dipped in 50%, 70% and 95% ethanol and xylene. After sections were covered coverslip with mounting solution (Sigma). The thickness of the epidermis and the COX‐2 were visualized using a fluorescent microscope (Leica microsystems,) and images were analysed using Leica Application Suite X (Leica microsystems) software.

### 2.13 Statistical analysis

Where appropriate, data are expressed as the mean ± standard deviation (SD), and significant differences between UVB and 4‐PP groups were calculated with one‐way anova with LSD *post‐hoc* test (analysis of variance) of IBM SPSS software (IBM Corp.,). Student's *t*‐test was used to assess significance of differences between the groups. A probability value of *p* < 0.05 was used as the criterion for statistical significance.

## 3 RESULTS

### 3.1 4‐PP inhibits UVB‐induced COX‐2 expression, PGE_2_ production and AP‐1 transactivity in HaCaT cells

**FIGURE 1:**
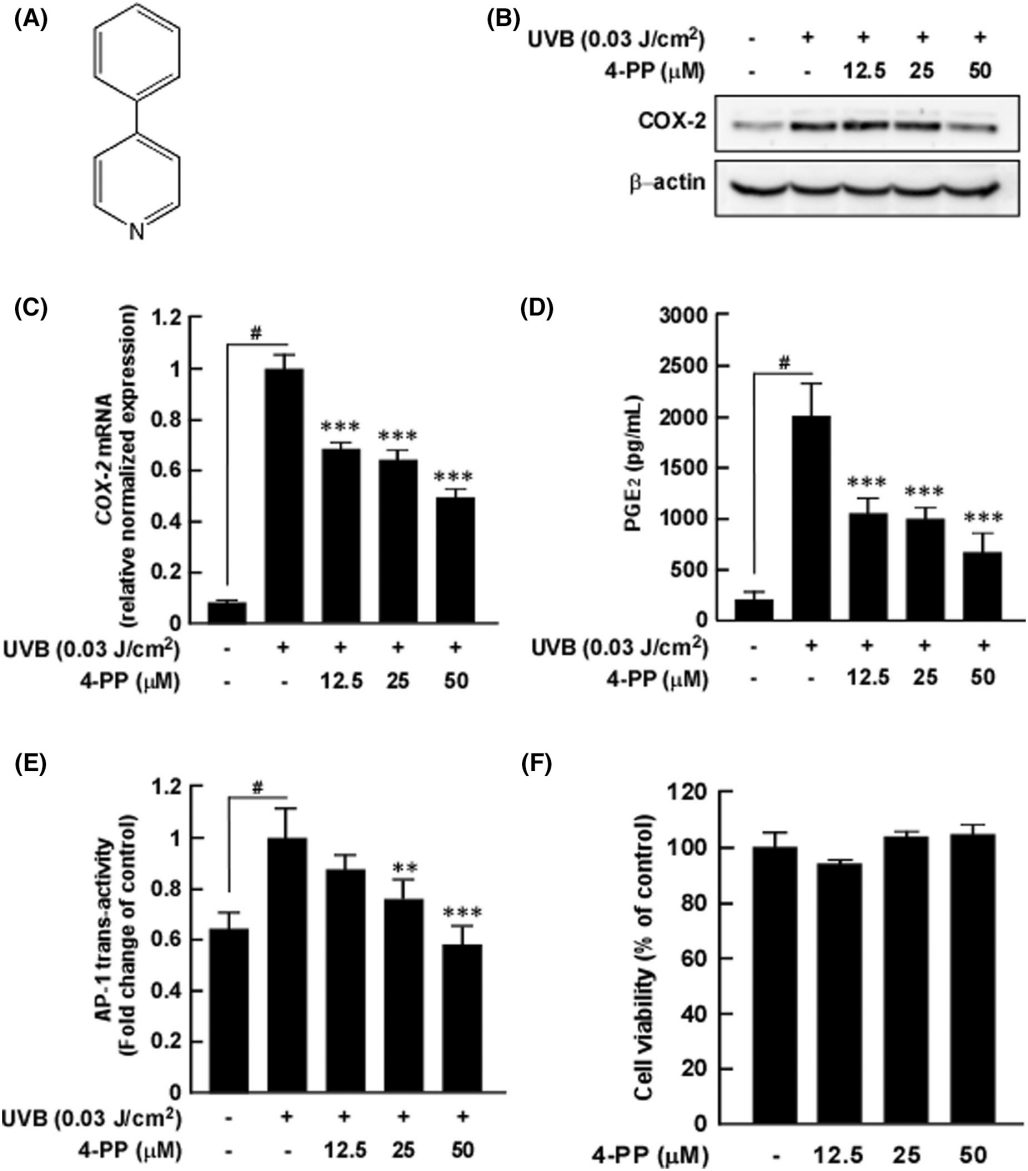
The effect of 4‐phenylpyridine (4‐PP) on UVB‐induced COX‐2 protein and *COX‐2* mRNA expression, PGE_2_ production, AP‐1 transactivity and cell viability in HaCaT cells. (A) Chemical structure of 4‐PP. (B) and (C) 4‐PP inhibits UVB‐induced COX‐2 expression and *COX‐2* mRNA expression in HaCaT cells. Protein and mRNA levels were analysed by western blot assay and RT‐qPCR, respectively. (D) 4‐PP inhibits UVB‐induced PGE_2_ production in HaCaT cells. Expression levels of PGE2 were determined using Prostaglandin E_2_ ELISA kit. (E) 4‐PP inhibits UVB‐induced AP‐1 transactivity in HaCaT cells. HaCaT cells were stably transfected with an AP‐1‐luciferase plasmid. Cells were then treated with 4‐PP for 1 h prior to UVB exposure and then incubated for 5 h. (F) 4‐PP was non‐toxic to HaCaT cells at 12.5, 25 and 50 μM. Cell viability was measured by the MTS assay. The data represent the mean ± SD of three independent experiments. #*p* < 0.05 between the control group and the group exposed to UVB alone; **p < 0.01 and ***p < 0.001 between groups irradiated with UVB and 4‐PP and the group exposed to UVB alone

COX‐2 is a critical factor in UV‐caused skin inflammation and could be an effective target for regulation of skin diseases.^15^ Therefore, we first investigated the effect of 4‐PP on UVB‐induced COX‐2 expression in HaCaT cells. 4‐PP significantly inhibited UVB‐induced COX‐2 expression and *cox‐2* mRNA in HaCaT cells (Figure 1B,C). PGE_2_, a product of COX‐2, induces acute inflammation and inflammatory disorders.^16^ 4‐PP significantly inhibited UVB‐induced PGE_2_ production in HaCaT cells (Figure 1D). Additionally, we observed that 4‐PP significantly inhibited UVB‐induced AP‐1 activity in HaCaT cells (Figure 1E). 4‐PP was non‐toxic at 12.5, 25 and 50 μM in HaCaT cells (Figure 1F).

### 3.2 4‐PP inhibits UVB‐induced phosphorylation and translocation of c‐Jun in HaCaT cells

**FIGURE 2:**
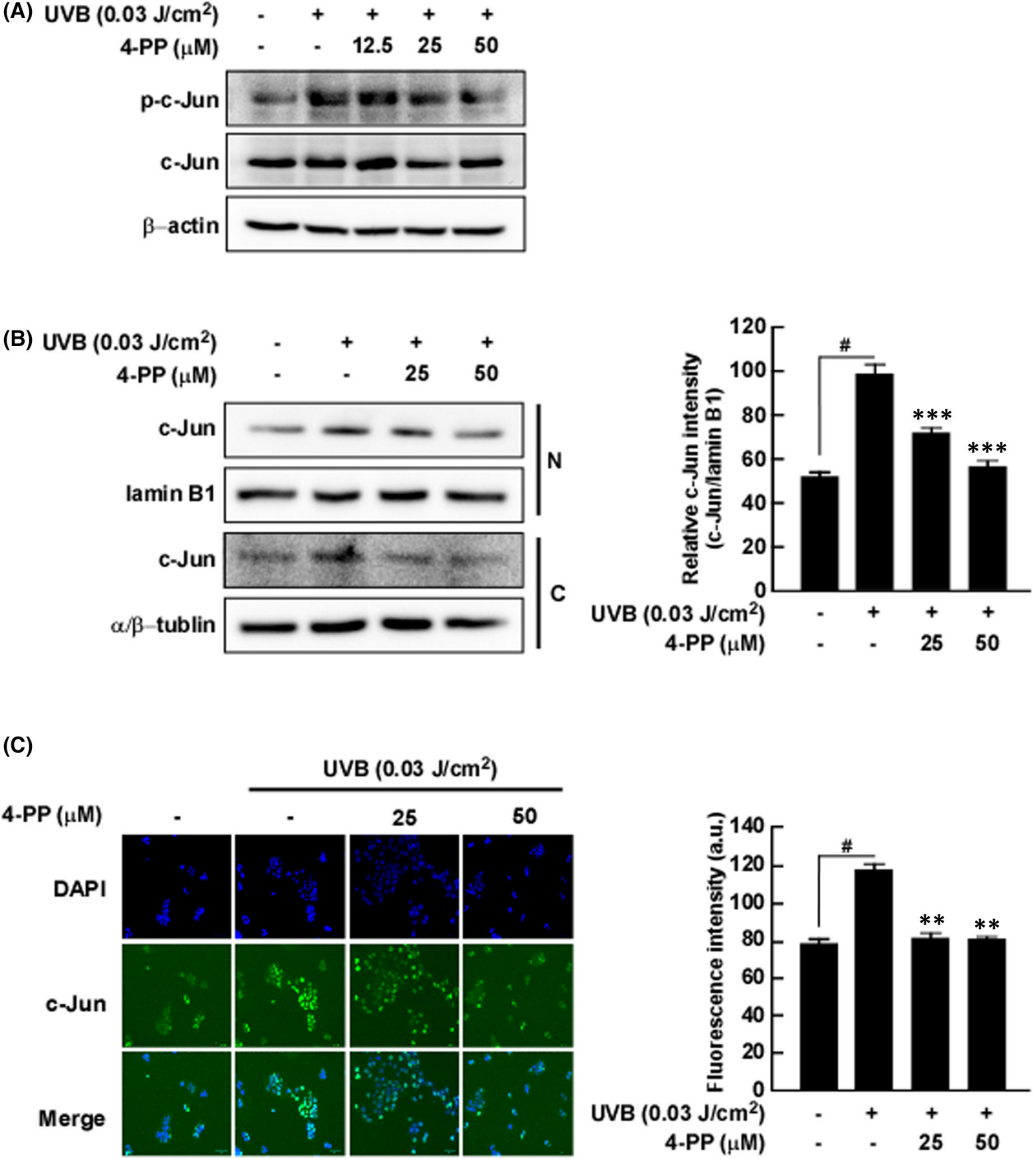
The effect of 4‐PP on UVB‐induced phosphorylation and nuclear translocation of c‐Jun in HaCaT cells. (A) 4‐PP inhibits UVB‐induced phosphorylation of c‐Jun in HaCaT cells. (B) and (C) 4‐PP inhibits UVB‐induced translocation of c‐Jun in HaCaT cells; N, nuclear fractions; C, cytosol fractions. Cells were pretreated with 4‐PP for 1 h, treated with UVB and harvested after 30 min. Phosphorylation of c‐Jun in whole lysate and c‐Jun expression in the nuclear and cytoplasmic fractions was analysed by western blot assay. Cytosolic and nuclear fractions and immunofluorescence were described in the Materials and Methods section. The data represent the mean ± SD of three independent experiments. #*p* < 0.05 between the control group and the group exposed to UVB alone; **p < 0.01 and ***p < 0.001 between groups irradiated with UVB and 4‐PP and the group exposed to UVB alone

The COX‐2 promoter contains several binding sequences for various transcription factors including AP‐1 and contains c‐Jun and c‐Fos subunits.^17^ To confirm whether the inhibition of COX‐2 expression by 4‐PP was related to AP‐1 activity, we measured phosphorylation and translocation of c‐Jun using western blot assay and immunofluorescence. 4‐PP inhibited UVB‐induced phosphorylation of c‐Jun in HaCaT cells (Figure 2A). Western blot assay and immunofluorescence assay results demonstrated that 4‐PP significantly reduced UVB‐induced translocation of c‐Jun from cytoplasm to nucleus in HaCaT cells (Figure 2B,C, respectively).

### 3.3 4‐PP inhibits UVB‐induced phosphorylation of MAPKKs/MAPKs in HaCaT cells

**FIGURE 3:**
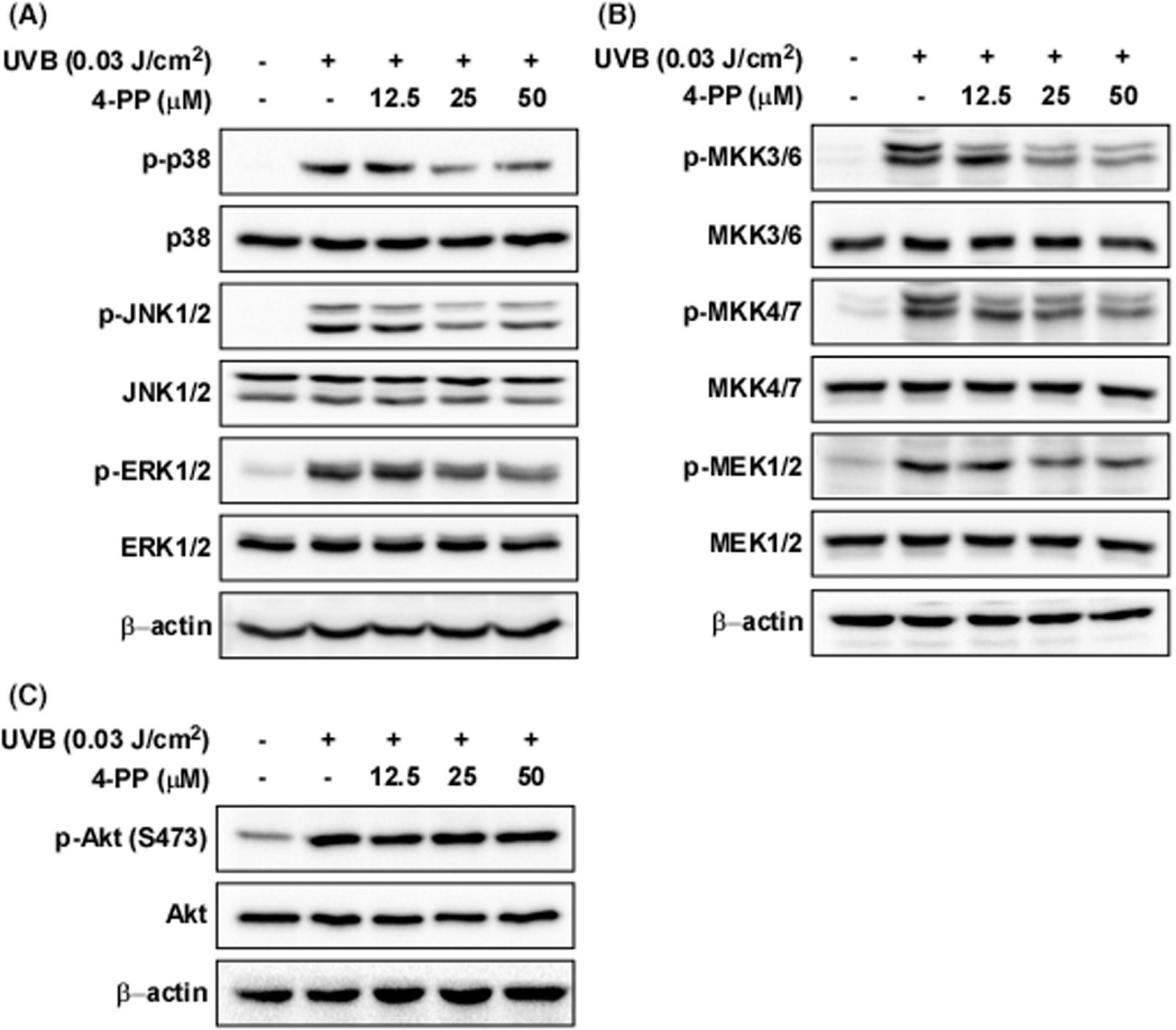
The effect of 4‐PP on UVB‐induced phosphorylation of MAPKs and MAPKKs in HaCaT cells. (A) 4‐PP inhibits UVB‐induced phosphorylation of p38, JNK1/2 and ERK1/2 and in HaCaT cells. (B) 4‐PP inhibits UVB‐induced phosphorylation of MKK3/6, MKK4/7 and MEK1/2 in HaCaT cells. (C) 4‐PP did not affect the phosphorylation of Akt. Cells were pretreated with 4‐PP for 1 h, treated with UVB and harvested after 30 min. Phosphorylation and expression levels were detected by western blot assay with specific antibodies

MAPKs are representative signalling molecules in the regulation of UV‐medicated AP‐1 transcriptional activity.^18^ and therefore we evaluated the effect of 4‐PP on UVB‐induced phosphorylation of MAPKs in HaCaT cells. 4‐PP reduced UVB‐induced phosphorylation of p38, JNK1/2 and ERK1/2 in HaCaT cells (Figure 3A). We also evaluated the effect of MAPKKs, the upstream kinase of MAPKs, as 4‐PP affect phosphorylation of MAPKs. The results showed that 4‐PP suppressed UVB‐induced phosphorylation of MKK3/6, MKK4/7 and MEK1/2 in HaCaT cells (Figure 3B). However, 4‐PP did not affect the phosphorylation of Akt (Figure 3C).

### 3.4 4‐PP inhibits UVB‐induced phosphorylation of EGFR by direct binding to c‐Src in HaCaT cells

**FIGURE 4:**
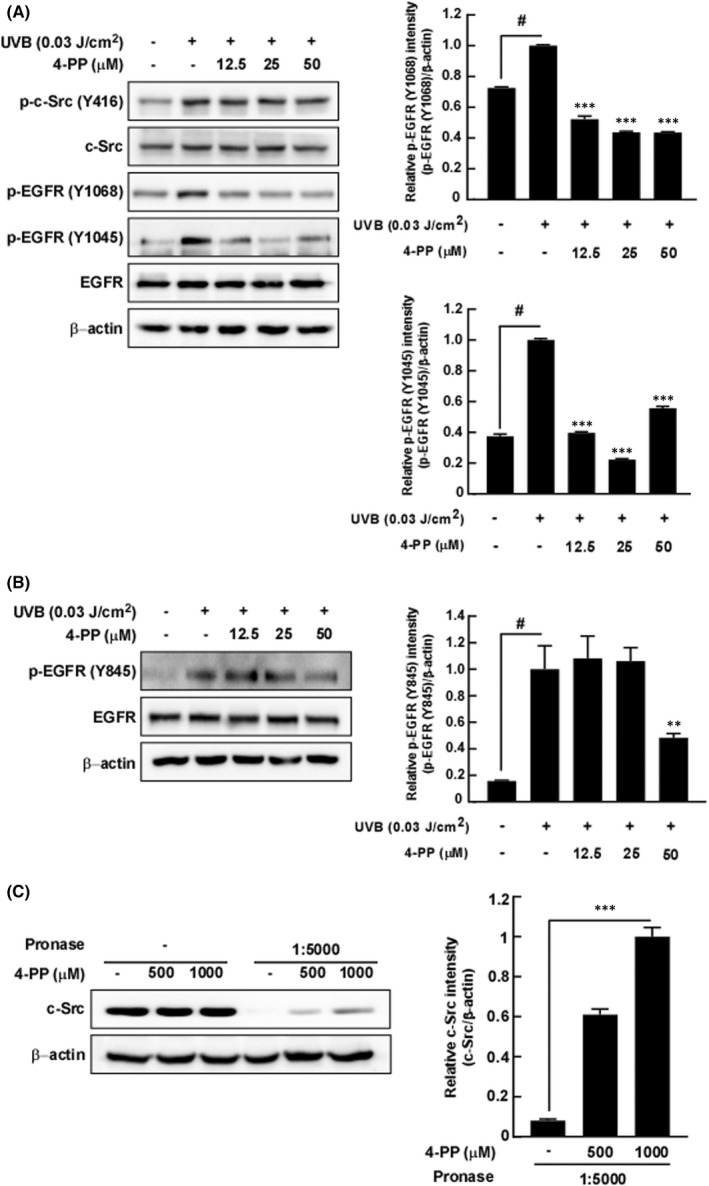
The effect of 4‐PP on the UVB‐induced phosphorylation of c‐Src and EGFR in HaCaT cells. (A) 4‐PP inhibits the UVB‐induced phosphorylation, EGFR (Y1068) and EGFR (Y1045) in HaCaT cells, but had no effect on phosphorylation of c‐Src. (B) 4‐PP inhibits the UVB‐induced phosphorylation of EGFR (Y845) in HaCaT cells. Cells were pretreated with 4‐PP for 1 h, irradiated with UVB, and then harvested after 15 min. Phosphorylation and expression levels were detected by western blot assay with specific antibodies. The data represent the mean ± SD of three independent experiments. #*p* < 0.05 between the control group and the group exposed to UVB alone; **p < 0.01 and ***p < 0.001 between groups irradiated with UVB and 4‐PP and the group exposed to UVB alone. (C) 4‐PP directly binds to c‐Src in HaCaT cells. For the DARTS assay, cells lysate was treated with 4‐PP at the indicated concentrations for 1 h and were digested with pronase and assessed via western blot assay. The data represent the mean ± SD of three independent experiments. **p < 0.01 between groups treated with pronase and 4‐PP and the group treated with pronase only

Phosphorylation of MAPKKs and MAPKs was suppressed by 4‐PP; therefore, we evaluated the effect of 4‐PP on upstream kinases of MAPKKs such as c‐Src and EGFR. Interestingly, although 4‐PP did not affect the UVB‐induced phosphorylation of c‐Src, UVB‐induced phosphorylation of EGFR at Tyr 1068 and EGFR Tyr 1045 in HaCaT was suppressed by 4‐PP. (Figure 4A). c‐Src is known to regulate EGFR activity by phosphorylation of EGFR at Tyr 845.^19^ Therefore, we measured the effect of 4‐PP on UVB‐induced phosphorylation of EGFR at Tyr 845 and demonstrated that 4‐PP inhibited phosphorylation at this residue (Figure 4B). Since 4‐PP inhibits UVB‐induced activation of c‐Src‐specific downstream regulator, we hypothesized that 4‐PP may affect EGFR activity via direct binding to c‐Src. To confirm our hypothesis, we conducted a DARTS assay and revealed that 4‐PP increased c‐Src stability against pronase treatment (Figure 4C).

### 3.5 Knockdown of c‐Src inhibits UVB‐induced COX‐2 expression and phosphorylation of MAPKs and EGFR in HaCaT cells

**FIGURE 5:**
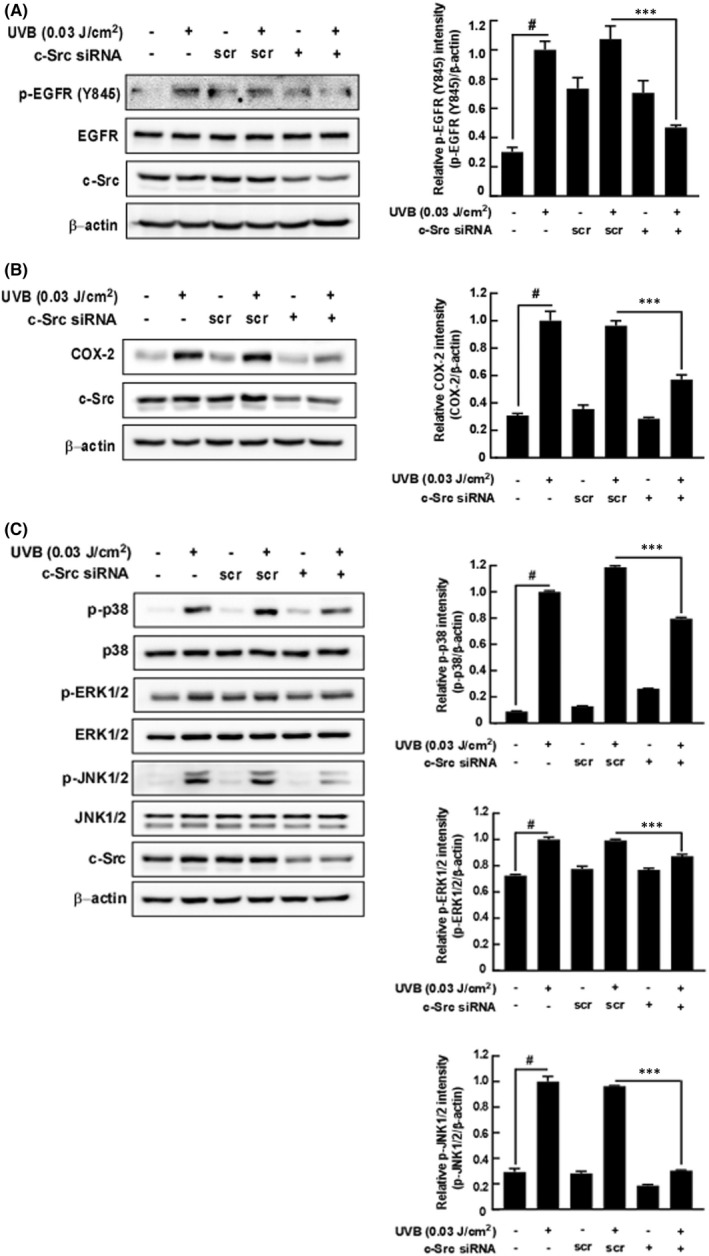
The effect of c‐Src knockdown on the UVB‐induced phosphorylation of MAPKs and COX‐2 expression in HaCaT cells. (A) Knockdown of c‐Src inhibits the UVB‐induced phosphorylation of EGFR at Tyr845 in HaCaT cells. (B) Knockdown of c‐Src inhibits the UVB‐induced COX‐2 expression in HaCaT cells. (C) Knockdown of c‐Src inhibits the UVB‐induced MAPKs signalling pathway in HaCaT cells; scr, scrambled siRNA. siRNA knockdown assay was described in the Materials and Methods section. The data represent the mean ± SD of three independent experiments. #*p* < 0.05 between the control group and the group exposed to UVB alone;**p < 0.01 and ***p < 0.001 between groups treated with scrambled siRNA and UVB and groups treated with c‐Src siRNA and UVB

To confirm the role of c‐Src on UVB‐induced COX‐2 expression and MAPKs signalling pathways, we transfected HaCaT cells with siRNA for c‐Src knockdown. Knockdown of *c‐Src* mRNA inhibited UVB‐induced phosphorylation of EGFR at Tyr845 (Figure 5A). Additionally, Knockdown of c‐Src inhibited UVB‐induced COX‐2 expression (Figure 5B) and phosphorylation of MAPKs including p38, ERK1/2, JNK1/2 (Figure 5C).

### 3.6 4‐PP inhibits UVB‐induced epidermal thickness, COX‐2 expression and phosphorylation of EGFR (Y845) in ICR mice

**FIGURE 6:**
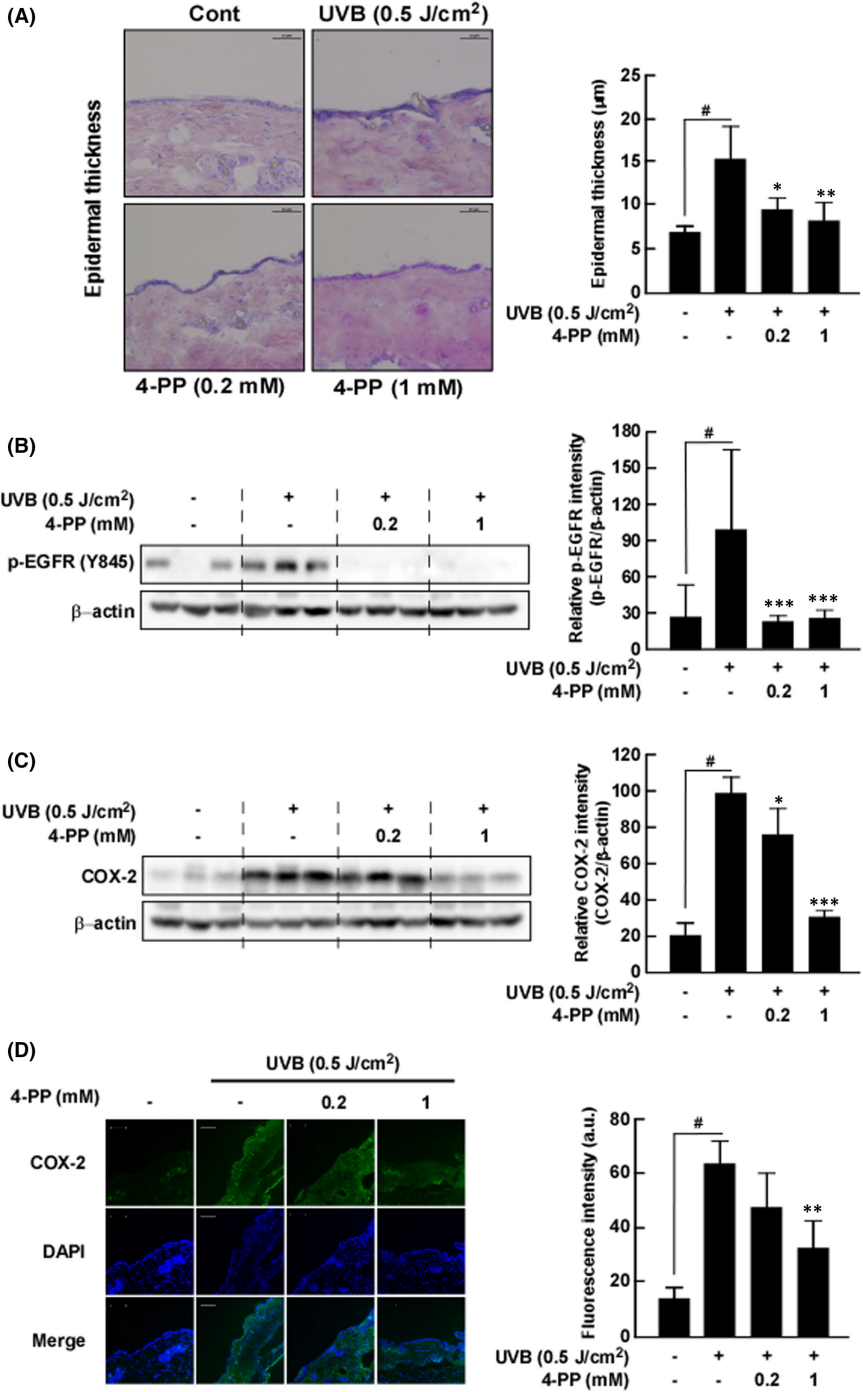
The effect of 4‐PP on UVB‐induced epidermal thickness, phosphorylation of EGFR (Y845) and COX‐2 expression in ICR mice. (A) 4‐PP inhibits UVB‐induced increasing mouse epidermal thickness. Haematoxylin and eosin‐stained images of UVB‐irradiated mouse skin. Images are representative of results from 5 tissue samples. (B) 4‐PP inhibits UVB‐induced phosphorylation of EGFR (Y845) in ICR mice. (C) and (D) Western blot assay and immunofluorescence assay show that 4‐PP inhibits UVB‐induced COX‐2 expression in ICR mice. Phosphorylation and expression levels of mice dorsal skin were detected by western blot assay. The data represent the mean ± SD of three independent experiments. ^#^ *p* < 0.05 between the control group and the group exposed to UVB alone; *p < 0.05, **p < 0.01 and ***p < 0.001 between groups irradiated with UVB and 4‐PP and the group exposed to UVB alone

We then measured the inhibitory effect of 4‐PP on UVB‐induced skin inflammation in vivo. 4‐PP treated mice showed a significant reduction in epidermal thickening compared to the UVB irradiation‐only mice (Figure 6A). Western blot assay with protein extract from mouse skin showed that UVB irradiation of the dorsal skin of mice up‐regulated phosphorylation of EGFR (Y845) and COX‐2 expression and that this abnormal expression was significantly suppressed by 4‐PP (Figure 6B,C). Additionally, immunofluorescence results also showed that treatment of 4‐PP suppressed UVB‐induced COX‐2 expression in mouse skin (Figure 6D).

## 4 DISCUSSION

The epidermis of skin enables essential photoprotection, adaptive immunity and absorption of harmful chemicals.^20^ UV light is key harmful environmental factor and causes skin inflammation and development of skin cancer.^21^ Skin damage caused by UV exposure is important as a pre‐stage of disease development, and can also cause anxiety due to effects on physical appearance.

Recently, cosmeceuticals, cosmetic with added pharmacological efficacy, have received increased attention.^22^ Previously, we proved that natural compounds could be promising cosmeceutical materials with preventive capacity for UVB‐induced skin inflammation and skin cancer.^5^, ^8^ Cruciferous vegetables are promising anti‐inflammatory agents, and we previously screened 30 cruciferous compounds via UVB‐induced COX‐2 expression in HaCaT cells. Among them, we selected 4‐PP as the most potent anti‐skin inflammatory agents candidate (Data not shown). 4‐PP is a compound found in Chinese cabbage and orange oil. Berteroin,^23^ caffeic acid and sinapic acids^24^ from Chinese cabbage have been studied for anti‐inflammatory effects. However, the effect of 4‐PP on UVB‐induced skin inflammation and its molecular target(s) remain unclear.

COX‐2 is considered as an inflammatory factor in skin damage with increased expression in response to UV exposure and is closely related to skin inflammation and finally development of skin cancer.^25^ Therefore, we examined the effect of different concentrations of 4‐PP (12.5–50 μM) on UVB‐induced *COX‐2* mRNA and protein levels in HaCaT cells. 4‐PP significantly suppressed UVB‐induced *COX‐2* mRNA and protein expression in these cells. AP‐1 transcription factor, which comprises a heterodimer of c‐Jun and c‐Fos and a homodimer of c‐Jun and c‐Jun, has a central role in UV‐induced *COX‐2* gene expression in HaCaT cells^26^ and moves from the cytosol to the nucleus to promote gene expression. We confirmed that the phosphorylation of c‐Jun was increased by UV irradiation and was inhibited by 4‐PP. Western blot assay and immunofluorescence results also showed that 4‐PP significantly suppressed c‐Jun translocation from cytosol to the nucleus. These results indicate that 4‐PP suppressed UVB‐induced COX‐2 expression by regulation of c‐Jun phosphorylation and translocation from cytosol to the nucleus.

Activated MAPKs leads to AP‐1 activation by enhancing the expression and nuclear translocation of AP‐1 subunits and their binding to DNA‐responsive elements.^27^ MAPK is phosphorylated by several MAPKKs, such as ERK‐MEK1/2, JNK‐MKK4/7 and P38‐MKK3/6.^28^ We confirmed that 4‐PP inhibits UVB‐induced phosphorylation of MAPKKs and MAPKs without affecting Akt in HaCaT cells. Since the MAPKKs/MAPKs signalling pathway was inhibited, we further investigated the effect of 4‐PP on UVB‐induced receptor tyrosine kinase and related kinases, such as EGFR and c‐Src. Interestingly, 4‐PP inhibited phosphorylation of EGFR (Y1068) and EGFR (Y1045) but not c‐Src. Tyrosine 1068 and 1045 in EGFR are known as auto‐phosphorylation sites. Moreover, several studies reported that active c‐Src directly phosphorylates EGFR at tyrosine 845.^29^ In our western blot assay, we found that 4‐PP inhibited UVB‐induced phosphorylation of EGFR at tyrosine 845; however, 4‐PP did not affect UVB‐induced phosphorylation of c‐Src, and therefore we hypothesized that 4‐PP directly affects phosphorylation of EGFR at tyrosine 845 by direct binding to c‐Src. The DARTS assay can be used to prove the interaction between compound and target protein by using a proteinase, such as pronase.^30^ Using the DARTS assay, we evaluated the interaction between 4‐PP and c‐Src and found that 4‐PP directly bound to c‐Src. We then investigated how 4‐PP affects phosphorylation of EGFR and subsequent MAPKKs/MAPKs signalling pathways after interacting with c‐Src. Additionally, knockdown of c‐Src suppressed UVB‐induced phosphorylation of EGFR at Tyr 845 and MAPKs and expression of COX‐2. A previous study suggested that c‐Src regulates UVB‐induced EGFR phosphorylation at tyrosine 845, MAPKs, Akt and expression of COX‐2 in JB6P+ cells.^10^ Our results indicated that 4‐PP binds to c‐Src and inhibits c‐Src interaction with EGFR and subsequently suppressed MAPKKs/MAPKs/AP‐1/COX‐2 signalling pathways.

To verify the effect of 4‐PP on UVB‐induced skin inflammation, we performed in vivo experiments. We found that acute UVB irradiation to the dorsal skin of mice increased COX‐2 expression, whereas pretreatment of 4‐PP prevented the UVB‐induced abnormal COX‐2 expression in mouse skin. Additionally, pretreatment of 4‐PP significantly suppressed UVB‐induced EGFR phosphorylation of EGFR at Tyr 845 in mouse skin.

## 5 CONCLUSION

In this study, we confirmed that 4‐PP, a compound found in Chinese cabbage and orange oil, inhibits UVB‐induced COX‐2 expression through inhibition of EGFR/MAPKKs/MAPKs signalling pathways by targeting c‐Src in HaCaT cells. Additionally, we observed dorsal treatment of 4‐PP suppressed EGFR phosphorylation of EGFR tyrosine 845 and COX‐2 expression in ICR mice. Overall, these results reveal that 4‐PP could be a promising anti‐inflammatory agent by preventing UVB‐mediated skin inflammation.

## AUTHOR CONTRIBUTIONS

**Ju Gyeong Kim:** Data curation (lead); formal analysis (lead); investigation (lead); methodology (equal); validation (equal); writing – original draft (lead); writing – review and editing (equal). **Ha Yeong Kang:** Data curation (equal); formal analysis (equal); investigation (equal); visualization (equal). **Min Jeong Kim:** Data curation (equal); investigation (equal); writing – review and editing (equal). **Seokwon Lim:** Software (equal); validation (equal). **Chang Joo Lee:** Project administration (equal); software (equal). **Kyung‐Min Kim:** Funding acquisition (equal). **Sung Keun Jung:** Conceptualization (equal); funding acquisition (equal); methodology (equal); project administration (equal); supervision (lead); validation (equal); writing – original draft (equal); writing – review and editing (lead).

## CONFLICT OF INTEREST

There is no conflict of interest.

## Data Availability

The data that support the findings of this study are available from the corresponding author upon reasonable request.
